# Case Reports of *Situs Inversus Totalis* and Dextrocardia in Sprague Dawley Rats

**DOI:** 10.3390/vetsci6030067

**Published:** 2019-08-15

**Authors:** Reynaldo Oliva Hernández, Jordan D. Lewicky, Nya L. Fraleigh, Hoang-Thanh Le

**Affiliations:** 1 Finlay Institute of Vaccine Research and Production, Havana 11600, Cuba; 2 Health Sciences North Research Institute (HSNRI), Sudbury, ON P3E2H3, Canada; 3 Northern Ontario School of Medicine (NOSM), Sudbury, ON P3E2C6, Canada; 4 Chemistry & Biochemistry and Biology Departments, Laurentian University, Sudbury, ON P3E2C6, Canada

**Keywords:** *Situs inversus totalis*, dextrocardia, Sprague Dawley rats

## Abstract

*Situs inversus totalis* is a condition where there is a transposition of all internal organs from their normal anatomical location. This infrequent and rare congenital condition has been described in several species of mammals. Dextorcardia is a series of conditions associated with an abnormal congenital positioning of the heart, and is often associated with *situs inversus totalis*. Here we report a case of *situs inversus totalis* and two cases of dextrocardia identified in Sprague Dawley rats during gross necropsy evaluations at both the Health Sciences North Research Institute (HSNRI) in Canada and Finlay Institute of Vaccine Research and Production in Cuba. The intent of this report is to share our findings and aid in the accumulation of data on these rare conditions.

## 1. Introduction

*Situs inversus totalis* (SIT) is a rare congenital malformation characterized by the complete reversal (transposition) of the normal location of the thoracic and abdominal organs [1]. SIT is a very rare condition, with the incidence rate in humans estimated to be 0.01–0.02% [2], within which there is an equal prevalence among males and females [3]. The exact causes of SIT are unknown. As the mechanisms for asymmetry in vertebrates are being defined, a number of genes that express asymmetry in embryonic development are being identified that appear to play a critical role in determining the left–right axis and may be linked to the development of SIT [4]. SIT does not typically affect quality of life or lifespan; however, it has been linked to cardiac and vascular alterations associated with increased risks of heart, spleen and hepatobiliary malformations [5,6]. SIT is also associated with Kartagener’s syndrome, which is an autosomal recessive disorder also known as primary ciliary dyskinesia [7].

Dextrocardia is a broadly encompassing term for any congenital abnormality in the positioning and orientation of the heart [8]. The overall incidence rate of dextrocardia in humans has been difficult to accurately determine given that it is generally asymptomatic, and seems to be identified more among those with some form of cardiac condition [9]. The majority of dextrocardia cases reported are associated with SIT, and in particular Kartagener’s syndrome [8]. Dextrocardia is associated with various heart malformations and malfunctions, but in general these associations are less when part of SIT [8].

In addition to humans, there are reports of SIT and dextrocardia in different species of animal, both domestic and wild, including fish [10], pigs [11], cats [12], dogs [13,14,15], horses [16], and rodents [17,18,19]. Within the latter, these anatomical alterations have been described in both Wistar and Sprague Dawley (SD) rats [17,18]. The overall incidence rate of SIT in rats in higher than in humans, and has been estimated to be 0.01–0.20% [19]. While the overall incidence of SIT in rats is higher than in humans [19], case reports on rats occur less frequently than for either humans or other animals. This paper presents a case of SIT and two cases of dextrocardia in SD rats discovered during independent vaccine evaluation studies. These reports will be helpful in the accumulation of data on these rare conditions in rats.

## 2. Materials and Methods

Studies done at HSNRI involved female SD rats (Charles River, Montreal, QC, Canada). The animals were housed in Innocage^®^ rat cages at the Animal Care Facility at Laurentian University. Interior dimensions: 141 square inch floor space, 7” height (909 cm^2^ floor space, 17.8 cm height), outside dimensions: 17” L × 13.4” W × 7.8” H (maximum), 43.2 × 34.0 × 19.8 cm (maximum), 100% PET plastic and BPA-Free. Rats were provided specialized rodent feed (Teklad 8640 22/5, Envigo, Mississauga, ON, Canada), and Aquavive^®^ acidified rat water was provided in bottles, which were both available *ad libitum*. The animal room was maintained at a temperature of 21 ± 2 °C and a relative humidity of 55 ± 5%. All protocols were approved by the Animal Care Committee at Laurentian University (Protocol #: 2016-09-02) and the Biosafety Committee at Health Sciences North Research Institute.

Studies done at the Finlay Institute involved male rats (Centro Nacional para la Producción de Animales de Laboratorio (CENPALAB), Havana, Cuba). The animals were housed in Tecniplast^®^ rat cages at the Animal Care Facility at the Finlay Institute of Vaccine Research. Dimensions and model: 1354 G Eurostandard Type IV, 595 × 380 × 200 mm, floor area 1820 cm, PEI plastic and BPA-Free. Rats were provided specialized rodent feed (EMO 1002, ALYco^®^, CENPALAB), and acidified water (2.5–2.7 pH) was provided in bottles (750 mL volume). Both food and water were available *ad libitum*. The animal room was maintained at a temperature of 22 ± 2 °C and a relative humidity of 60 ± 5%. Blood hematology analysis was performed using an automatic analyzer (BC-2800, Mindray Medical International, Shenzhen, China) and serum chemistry was analyzed using a semi-automatic analyzer (BA-88A, Mindray Medical International, Shenzhen, China). All protocols were approved by the Laboratory Animal Care Committee (Protocol #: 16P03 and 17P01) and the Biosafety Committee at Finlay Institute of Vaccine Research and Production.

## 3. Results

### 3.1. Case 1

**Figure 1:**
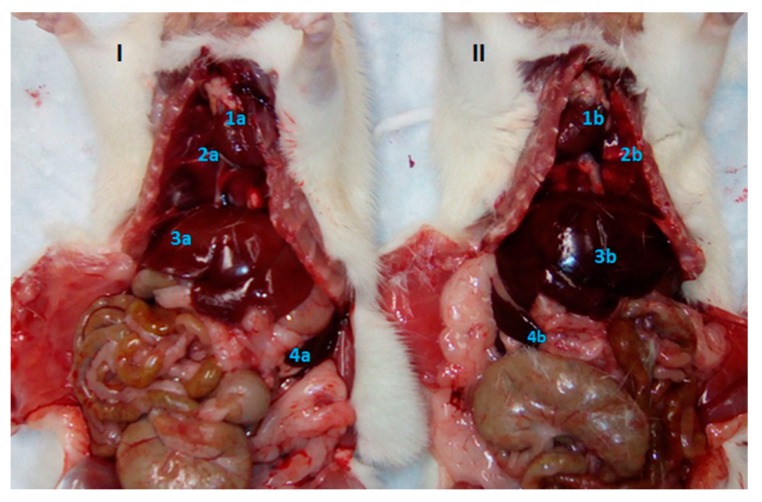
Macroscopic observation of organs in situ in Sprague Dawley rats. (**I**) Rat with organs located in normal positions. (**II**) Rat with *Situs inversus totalis* (SIT) and organ transposition. (1-) Heart, (2-) Lungs, (3-) Liver, (4-) Spleen. (a-) Correct position, (b-) Incorrect position.

This case describes an observation of SIT discovered during the immunological evaluation of a novel nicotine vaccine developed by Dr. Hoang-Thanh Le and his team at the Health Sciences North Research Institute in Sudbury, Ontario, Canada. Routine pathological investigations were carried out on a group of female SD rats that were approximately 34 weeks of age at the time of euthanasia. SIT was identified incidentally in a single rat during gross necropsy. Macroscopic observations of the thoracic and abdominal cavities showed translocation of organs, including the heart, lungs, liver and spleen (Figure 1). Throughout the study the animal displayed no evidence or clinical signs of the condition. At the time of discovery, the animal was clinically healthy, with a normal size for its category/age, and no visible differences in comparison to the other animals being studied. There was no evidence of injury or any malfunction of a particular organ or system.

### 3.2. Cases 2 and 3

**Figure 2:**
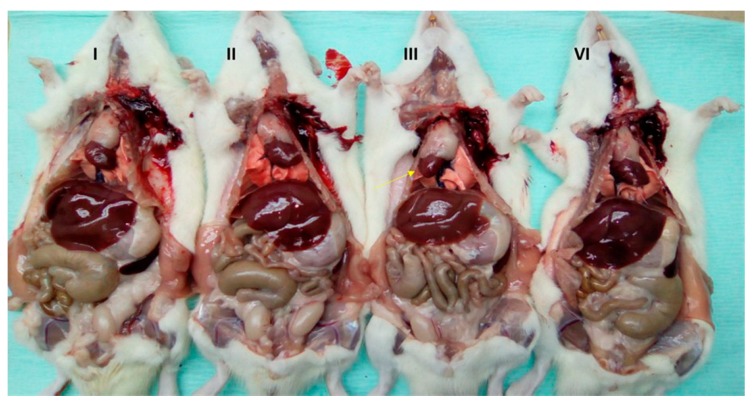
Macroscopic observation of organs in situ in Sprague Dawley rats. (**I**), (**II**), and (**IV**) Animals with the heart in correct position and central organs of the thoracic cavity to the left tip of the heart. (**III**) Animal with dextrocardia, arrow indicating the tip of the heart towards the right side.

**Figure 3:**
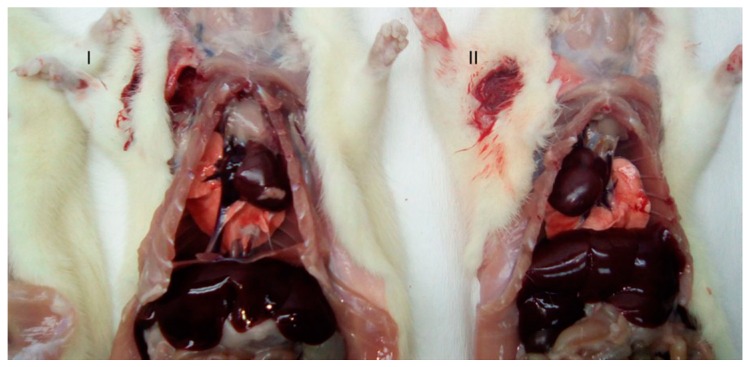
Macroscopic observation of organs in situ in Sprague Dawley rats. (**I**) Normal animal; (**II**) Animal with dextrocardia.

These cases describe two separate observations of dextrocardia discovered in male SD rats during preclinical toxicological evaluations at the Finlay Institute of Vaccine Research and Production in Havana, Cuba. Dextrocardia was incidentally identified in two separate rats (Figure 2 and Figure 3) during gross necropsy at different moments in time (6 months between Case 2 and Case 3). The dextrocardia was observed without transposition of any other organ; although the heart was found the right side and opposite its normal location, normal lung anatomy and situs was observed with the cardiac notch found on the left lung. A transposition of the aortic arch from its normal location in the center of the thoracic cavity was also observed. The animals displayed no evidence or clinical signs of the condition throughout the studies, and were clinically healthy at the time of discovery with weights/sizes within the normal ranges reported for the species and category (Case 2: 419.6 g, Case 3: 426.4 g, 14–16 weeks of age in each case). These anatomical abnormalities did not have any significant impact on various hematological parameters, serum chemistry, or organ weights (Table 1, Table 2 and Table 3)

## 4. Discussion

SIT and dextrocardia in humans is associated with a variety of other medical conditions, including Kartagener’s syndrome [5,6,7,14]. In animals, the association of these anatomical abnormalities with other medical conditions is far less prevalent, although SIT associated with Kartagener’s syndrome has been reported in dogs [7,14]. Both SIT and dextrocardia are generally asymptomatic conditions in humans and animals, usually only discovered during diagnostic imaging, surgical, or post-mortem necropsy procedures. As such, the number of cases of SIT and dextrocardia that go undiscovered may be quite substantial.

Here we report a case of SIT and two cases of dextrocardia observed in SD rats. In all three cases, the animals were healthy at the time of discovery, and displayed no evidence or clinical signs of the conditions. Our observation of SIT organ transposition in Case 1 (Figure 1) is not the first time that this condition has been reported in this species of rat [17]. These findings are interesting as they are the first reports of dextrocardia in laboratory animals in Cuba.

Our incidental discoveries of SIT and dextrocardia left us to determine if these anatomical abnormalities should be considered as exclusion criteria in our vaccine evaluation studies. In surveying the literature, there is no evidence to suggest that immunological and toxicological responses to a vaccine would be modified by the presence of SIT or dextrocardia when not associated with other medical conditions, nor would there be any impact of these anatomical abnormalities on organs at either the macroscopic or microscopic levels. The cases we reported agree with these findings, where other than the organ transpositions, there was no effect observed on any of the diagnostic parameters measured, including organ weights, as well as various serum chemistry and hematological parameters (Table 1, Table 2 and Table 3).

## 5. Conclusions

SIT and dextrocardia are rare congenital abnormalities that have been reported in humans and various other animal species. Typically, the conditions are asymptomatic and often go undiscovered. There is no evidence to support that immunological and toxicological responses to a vaccine or drug would be impacted by these anatomical abnormalities when not associated with other medical conditions. Case reports such as this will aid in the accumulation of data on these rare conditions.

## Figures and Tables

**Table 1:** Hematological studies of Sprague Dawley rats with dextrocardia.

Variable ^1^	Case 2	Case 3	Normal Range ^1^
Hemoglobin (g/L)	152	154	121–155
Hematocrit (mL/100 mL)	52.4	53.3	33.1–55.8
Leukocytes (10^3^/mm^3^)	5.6	5.5	4.8–13.0
Neutrophils (%)	11	13	4–13
Lymphocytes (%)	86	82	82–97

^1^ Values reported for SD rats of similar age from CENPALAB [20].

**Table 2:** Serum chemistry studies of Sprague Dawley rats with dextrocardia.

Variable ^1^	Case 2	Case 3	Normal Range ^1^
ATL (U/L)	66.5	63.9	32–81
AST (U/L)	129	150	89–216
ALP (U/L)	274	388	86–391
CPK (U/L)	1266	1841	233–4367
Creatinine (µmol/L)	73.7	66.5	40.7–77.8
Triglycerides (mmol/L)	0.93	0.63	0.51–1.35
Cholesterol (mmol/L)	1.50	1.16	1.04–2.19
Urates (µmol/L)	70.5	75.0	57.7–280.8
Urea (mmol/L)	8.8	9.1	8.3–15.8
Glucose (mmol/L)	9.0	10.0	5.2–10.9
Total Protein (g/dL)	5.6	5.3	5.5–7.2
Direct Bilirubin (mg/dL)	0.13	0.13	0.00–0.22

^1^ Values reported for SD rats of similar age from CENPALAB [20].

**Table 3:** Relative organ weight of Sprague Dawley rats with dextrocardia.

Variable	Relative Organ Weight ^1^ (%)	
	Case 2	Case 3
Brain	0.486	0.481
Heart	0.382	0.360
Thymus	0.100	0.102
Left lung	0.136	0.124
Right lung	0.240	0.238
Spleen	0.167	0.146
Liver	2.911	2.709
Left Kidney	0.376	0.312
Right Kidney	0.390	0.317

^1^ Relative weight is equal to organ weight multiplied by 100, divided by the end weight of the animal on the day of euthanasia.
